# Supplementary material for: Bayesian refinement of protein structures and ensembles against SAXS data using molecular dynamics
Source: PLoS Comput Biol. 2017 Oct 18;13(10):e1005800. doi: 10.1371/journal.pcbi.1005800 (PMC5662244; doi:10.1371/journal.pcbi.1005800)
Supplement: S1 Archive — (BZ2) [file pcbi.1005800.s011.tar.bz2 › gromacs_4.62_bayesian_saxs_refi/share/html/online/edo.html]

edo file format


## edo file format

|  |  |
| --- | --- |
| Main Table of Contents | **VERSION 4.6** |
| GROMACS homepage | **Sat 19 Jan 2013** |

---

### Description

Files with the edo file extension are generated by 
mdrun if Molecular Dynamics is performed with Essential Dynamics
constraints. Depending on the parameters set in the 
edi file, edo files may contain projections of positions,
velocities and forces onto selected eigenvectors during the run as well
as RMSD values, or information about specific types of constraints.
Specific results can be extracted from the edo files with standard unix
utilities like awk.

---

http://www.gromacs.org
